# Reinforcement of Gelatin-Based Nanofilled Polymer Biocomposite by Crystalline Cellulose from Cotton for Advanced Wound Dressing Applications

**DOI:** 10.3390/polym9060222

**Published:** 2017-06-13

**Authors:** Shukanta Bhowmik, Jahid M. M. Islam, Tonmoy Debnath, Muhammed Yusuf Miah, Shovon Bhattacharjee, Mubarak A. Khan

**Affiliations:** 1 Institute of Radiation and Polymer Technology, Bangladesh Atomic Energy Commission, Savar, Dhaka 1349, Bangladesh; shukantabd@gmail.com (S.B.); jahid.bmb@gmail.com (J.M.M.I.); 2 Department of Applied Chemistry and Chemical Engineering, Noakhali Science and Technology University, Noakhali 3814, Bangladesh; yuri19742003@yahoo.com (M.Y.M.); shovon_nstu@yahoo.com (S.B.); 3 Department of Public Health and Institute of Public Health, Chung Shan Medical University, Taichung City 40201, Taiwan; tonmoy1030@gmail.com; 4 Department of Microbiology, Noakhali Science and Technology University, Noakhali 3814, Bangladesh

**Keywords:** nanofilled composites, crystalline cellulose, gelatin, wound dressing, biocomposites

## Abstract

This study is designed to extract crystalline cellulose from cotton and reinforcing gelatin film for biomedical applications, especially as a wound dressing material for its exceptional biocompatibility and bio-activity. Moreover, gelatin helps in wound healing and crystalline cellulose as additive can improve its properties. Crystalline cellulose was prepared through hydrolysis and the effects of crystalline cellulose loading on the morphology, mechanical properties, and water sensitivity of the nanocomposite were investigated by means of scanning electron microscopy, tensile strength testing, and water absorption testing. Developed biocomposite film showed homogeneous dispersion of crystalline cellulose within the gelatin matrix and strong interfacial adherence between the matrix and reinforcement. Samples were tested for biocompatibility and in vitro cytotoxicity and found to have excellent biocompatibility without having any cytotoxicity. In vivo wound healing study in an animal model showed 40% increased healing than the model dressed by conventional dressing.

## 1. Introduction

Wound care is one of the major healthcare concerns worldwide. An advanced wound dressing can minimize suffering and treatment charge by reducing the rehabilitation period. Many researchers have reported techniques to develop advanced wound dressings having suitable infection barriers, wound exudate absorption, water-vapor permeability, as well as other features [1,2,3]. Although there are several commercially-available products, none are adequate and economically friendly. Therefore, the development of a simple, but effective, wound dressing material using gelatin can be fabricated by a simple process with an affordable cost, which is a basic requirement for developing and war-torn countries.

It is possible to derive crystalline rod-like particles from a variety of renewable sources, including natural fibers of plant sources, [4] wood, cotton, ramie, bacteria, and tunicates [5,6,7]. Natural fibers are being used as they are cheap, abundant, renewable, and biodegradable [8]. In recent years, nanocrystals have been used as fillers in composites due to their interesting low gas permeability [9,10,11,12] and stiffness-enhancing capacity [13]. They can also be used as reinforcements for adhesives, components of electronic devices, biomaterials, foams, aerogels, and textiles [14,15,16].

Gelatin is a film-formable, air-permeable, biocompatible, non-toxic, and haemostatic material that is beneficial for wound dressings [17]. Although gelatin is an important component of wound dressings [18], due to its poor mechanical properties and thermal instability, its use is restricted [19,20]. To improve the quality of gelatin, many researchers have incorporated various cross-linking reagents, such as glutaraldehyde [21], transglutaminase [22], plant polyphenols [23], formaldehyde [24], oxidized chondroitin sulfate [25], oxidized alginate [26], and dialdehydestarch [27]. However, no previous studies have focused on biocomposites of gelatin and natural cotton-derived nanocrystalline cellulose in wound dressings. Additionally, the available studies did not report more than 50% mechanical property enhancement, as well as increased stability in aqueous medium, which is expected through crystalline cellulose (CCs) reinforcement. In addition, only one study has focused on chitosan-gelatin microcapsules on cotton fabrics and its antibacterial effect [28]. Cellulose crystals comprise outstanding tensile strength and low density. It also has lower abrasion properties. Moreover, crystalline cellulose has a good dispersion ability in water [29]. Natural cotton is an excellent and cheaper source of cellulose which can be further converted to CCs for the reinforcement of several biobased matrices. Furthermore, using cotton-based CCs for the reinforcement of biocomposites may lead to recycling of cotton-based human-wares which is now a growing concern worldwide. In addition, CCs is biocompatible and biodegradable, hence it can be an excellent material to further studies. Thus, using this renewable material is a convenient, cost-effective approach to produce eco-friendly and sustainable products, as well as to conserve finite non-renewable natural resources.

Chengjun Zhou et al. reported an in situ polymerization process of polyacrylamide-cellulose nanocrystal composite hydrogels, CNCs accelerated the onset of gelation and acted as a multifunctional cross-linker during the gelation reaction as a result. The composite hydrogels exhibited enhanced steady-state elastic modulus and a plateau loss factor compared to those of the pure PAM (Polyacrylamide) hydrogels, indicating that adding CNCs not only reinforced, but also toughened, PAM hydrogels [30]. In addition, CCs are biocompatible and biodegradable, which can be essential to further studies. Therefore, in the present study, we extracted CCs from raw native cotton fromf Bangladesh which contains high amounts of cellulose. These CCs were then used to reinforce a gelatin composite film. Moreover, we investigated the reinforcing effect of CCs to gelatin-based biocomposite film by investigating various properties which were used for wound dressing in mice.

## 2. Experimental

### 2.1. Materials

Gelatin (Bloom Strength-240) of pharmaceutical grade was supplied by the Global Capsule Limited (GCL), Dhaka, Bangladesh. Cotton was collected from the pharmaceutical store of a local market. Sulfuric acid and sodium hydroxide were purchased from Merck, Darmstadt, Germany. All other reagents used were of analytical grade.

### 2.2. Methods

#### 2.2.1. Extraction of CCs

Twenty grams of commercial-grade raw cotton was cut into small pieces at about 2 cm lengths with scissors. Then the cut linter was put in a beaker filled with 1000 mL (10% *w*/*w*) NaOH. It was then boiled at 100 °C for 30 min to remove lignin from the cotton. This boiled cotton was washed until the complete removal of the NaOH solution. After that the cotton was rinsed with distilled water 2–3 times. After overnight drying at room temperature, the sample was weighed and acid hydrolysis was carried out by using 40% *w*/*w* H_2_SO_4_. This mixture was heated for 6 h at 55 °C. The extraction process is based on the fact that crystalline regions remain unsolved in acid solution; in other words, the disordered structure of the cellulose in the amorphous regions are sensitive to acid hydrolysis [31]. The solution was then neutralized with 10% NaOH solution and allowed to settle the cellulose layer. The supernatant was then collected and centrifuged for 6 h at 8000 rpm in a high-speed refrigerated centrifuge. Then a thick layer containing CCs was obtained at the bottom of test-tube. The CCs layer was collected by washing out the upper layer.

#### 2.2.2. Preparation of Gelatin Solution

Fixed amount (10% *w*/*v*) of pure gelatin solution was prepared by dissolving 10 g gelatin in 90 mL distilled water. The pH of the solution was around 4.5 and the resulted solution was placed on a hot plate with constant stirring with a magnetic stirrer wherein the solution was heated at 50–60 °C for 40–60 min.

#### 2.2.3. Preparation of Gelatin Film

The gelatin solution prepared by the above process was cast onto a releasing sheet (silicon cloth)-covered frame mounted on a flat glass plate for film formulation and then dried in an oven at 50 °C for 8 h at 45% relative humidity. The dried films (about 0.30 mm thickness) thus prepared were peeled off and cut into small pieces (50 mm × 10 mm) using conventional scissors.

#### 2.2.4. Preparation of CC/Gelatin Biocomposite Film

Three formulations were prepared at three ratios, where 2 mL, 5 mL, and 10 mL CCs were added with 100 mL gelatin solution. The resulting solutions were mixed with a magnetic hot plate stirrer at 40 °C for 30 min. Then they were further homogenized by sonication. Then the solutions were casted in a casting plate and dry CCs/gelatin composite films were obtained after one overnight drying.

### 2.3. Characterization

#### 2.3.1. Scanning Electron Microscopy (SEM)

External morphology (texture), crystalline structure, and orientation of materials in the samples were observed by a Philips XL30 scanning electron microscopy (SEM) at an accelerating voltage of 10 keV. Data were collected over a selected area of the surface of the sample, and a two-dimensional image was generated that displayed spatial variations.

#### 2.3.2. Fourier Transform Infrared Spectroscopy (FTIR)

Fourier transform infrared spectroscopy (FTIR) studies were conducted by modifying Vicentini et al. method [32]. To identify the functional groups present in the synthesized gelatin/CCs biocomposite samples, using a Prestige 21 (SHIMADZU) FTIR, Kyoto, Japan. The samples were oven-dried at 105 °C for 4–5 h, mixed with KBr in a ratio of 1:100 (*w*/*w*), and pressed under vacuum to form pellets. The FTIR spectrum of the samples was recorded in the transmittance mode in the range of 4000–400 cm^−1^ with an average of 30 scans. The resolution of the spectrometer was 4 cm^−1^.

#### 2.3.3. Mechanical Properties

Mechanical properties or tensile properties, such as tensile strength (TS), percent elongation at break (Eb), and tensile modulus (TM) of the biocomposite films, were measured by a Universal Testing Machine (Hounsfield, model H50 Ks 0404, Redhill, UK). The load capacity was 500 N and the efficiency was within ±1%. The speed of the tensile testing (crosshead speed) was 10 mm/min and the gauge length between two tensile grips was 40 mm. Fifty percent relative humidity at room temperature was maintained to enable an identical moisture content. All of the data regarding the mechanical properties are the average values of at least five individual readings and the percentage of accuracy is about ±1%.

#### 2.3.4. Buffer Uptake Properties

The experiment was designed to simulate an open exudating wound dressed with gelatin/CCs films. The water uptake of 10%, 5%, and 2%, CCs containing gelatin/CCs blended films, and pure gelatin/CCs blended film (control film) were measured at 50% relative humidity. The methodology was the same as that described by [33]. Briefly, a sponge was cut to fit into a 100 mL glass beaker to approximately 3/4 of its height. Phosphate buffer (0.1 N) was poured into the beaker containing the sponge. The sponge was squeezed and pulled to create a pumping action that accelerated the absorption of water. After the sponge was fully soaked, more buffer solution was added to a level of about 0.2 mm above the sponge top surface and the entire beaker placed in a water bath at 30 °C, the equilibrating condition cited from the U.S. Pharmacopoeia for transdermal delivery systems. A ca. 20 mm × 10 mm weighed samples was placed on the top surface of the soaked sponge. Only one side of the film was allowed to come into contact with the wet sponge surface. The sample was removed periodically, blotted dry with filter paper, and weighed until a constant weight was obtained. The Buffer uptake percentage, *W*_u_ by the sample was calculated from the expression:

Buffer uptake (*W*_u_) = (*W*_t_ − *W*_0_)/*W*_0_ × 100%

where, *W*_t_ = Weight after soaking, and *W*_0_ = Initial dry weight of the films.

#### 2.3.5. In Vitro Biocompatibility Analysis

Heparinized human blood was used to assay the biocompatibility of the developed Gelatin/CCs biocomposite film [34]. Samples were prepared using blood and gelatin/CCs biocomposite at ratios of 1:1, 2:1, and 4:1, respectively. A blood sample of the same donor was also diluted at the same ratios with distilled water and normal saline for control. After mixing the biocomposite, saline, and distilled water with blood at different ratios, the mixtures were kept in an incubator for 2 h at 37 °C. Then the samples were spread on glass slides and observed under a light microscope.

#### 2.3.6. Antimicrobial Property

The antimicrobial property of gelatin/CCs biocomposite film was identified against *Pseudomonas* sp. in nutrient agar medium by the disc diffusion method [35]. A streptomycin 10 µL standard antibiotic disk was used as a positive control. The blank disks (Oxoid, Hampshire, UK) were soaked in the sample solutions to prepare the working disc. One loop culture from the stock culture of *Pseudomonas* sp. was inoculated in normal saline and spread on a nutrient agar plate by sterile cotton buds. Then the working disc was introduced into the surface of the microorganism-inoculated agar plate at an appropriate spatial arrangement using ethanol-dipped and flamed forceps. The disk was pressed down to ensure complete contact with the agar surface. Plates were kept for 30 min for better absorption of the sample into agar media. Then the plates were placed in an incubator at 37 °C for 18 h. Inhibition zones were observed for understanding the antimicrobial activity.

#### 2.3.7. In Vitro Cytotoxicity Study

In vitro cytotoxicity testing was performed using the brine shrimp lethality bioassay method as described by Meyer et al., 1982, where rate of brine shrimp hatched and the number of death nauplii (larvae of brine shrimp) represents the strength of cytotoxicity of the sample [36]. Briefly, brine shrimps (*Artemiasalina*) were hatched using brine shrimp eggs in a conical-shaped vessel, filled with sterile artificial seawater and pH was adjusted at 8.5 using 1 N NaOH. The vessel was kept under constant aeration for 48 h. After hatching, active nauplii free from egg shells were collected from brighter portions of the hatching chamber and used for understanding the cytotoxicity. Composite film samples of 0.125, 0.25, 0.50, 0.75, 1.0 mg were dissolved in 1 mL artificial seawater, separately. Then the samples were put on Petri plates where the active nauplii were inoculated. After overnight incubation, the nauplii were counted. Vincristine sulfate (0.5 mg/mL) (an anticancer drug) was considered as the positive control.

#### 2.3.8. In Vivo Wound Healing

The wound-healing characteristics of gelatin/CCs biocomposite film was evaluated using a mouse model. All experiments were completed with the approval of the Bangladesh Atomic Energy Commission’s International Animal Care and Use Committee. Mice were anesthetized with 5 mL diethyl ether using an inhalation anesthesia system. The surgical area was shaved with an electric razor, the mice were strapped to a surgical board, and additional anesthesia was provided via a nose cone.

After a deep surgical plane of general anesthesia had been reached, a wound, approximately 1 cm in diameter and 1 mm in depth, was created on the left (lateral) side of the mice using curved blade surgical scissors. Both the epidermal and dermal layers were removed to create a full-thickness wound with minimal bleeding. Next, four diameters of the wound site were marked and measured using digital calipers and averaged to determine the original wound diameter and area. The wounded place was then dressed with one of three dressings: (1) conventional gauge bandage (control), (2) pure gelatin film and (3) gelatin/CCs biocomposite film.

The gelatin/CCs biocomposite and pure gelatin films were cut into 20 mm diameter circular sheets and were rehydrated with sterile normal saline immediately prior to use. The surgery was repeated three times on the same size of wound site in mice. Wound healing efficiency was measured by naked eye observation.

## 3. Result and Discussion

### 3.1. Morphology of CCs Derived from Cotton

**Figure 1:**
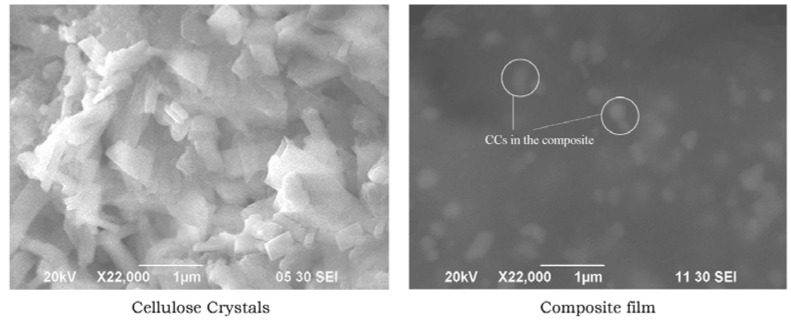
Scanning electron micrographs of cellulose crystals and gelatin/CCs (10:10) biocomposite film.

Size of CCs is very important for reinforcement as the longer the CCs is, the higher the stress transfer that will occur. Figure 1 presents SEM micrographs of the prepared CCs and the surface of the biocomposite film made of CCs extracted from raw cotton. The figure clearly represents well-isolated CCs from the cellulose microfibrils and the raw CCs was randomly oriented over the surface of the blended film belonging to the dilute regime (isotropic phase). The composite film showed etched features and appeared to have rough structures with many crystals present on the surface. The CCs appeared as white dots on the surface of the gelatin. As CCs is a very compacted structure, it can only bind with the gelatin by its outer surface. This may be why the whole CCs structure remains compacted and segregated in the gelatin CCs biocomposite and looks like white dots. To a certain extent, geometrical characteristics such as size, shape, and dimensions of cellulose nanocrystals depend on the nature of the cellulose source, as well as the hydrolysis conditions, such as time, temperature, ultrasound treatment, and purity of materials [7,37,38]. Here, diameter and size of fibrils of purified cellulose was reduced to a great extent due to the removal of all amorphous regions of semi-crystalline cellulose leaving nano-scale rod-like crystals. The extracted fibers were longer, with a length of 500 ± 100 nm. This value indicates a larger surface area of CCs prepared from cotton.

### 3.2. FTIR Analysis of Pure and Blend Biocomposite Films

**Figure 2:**
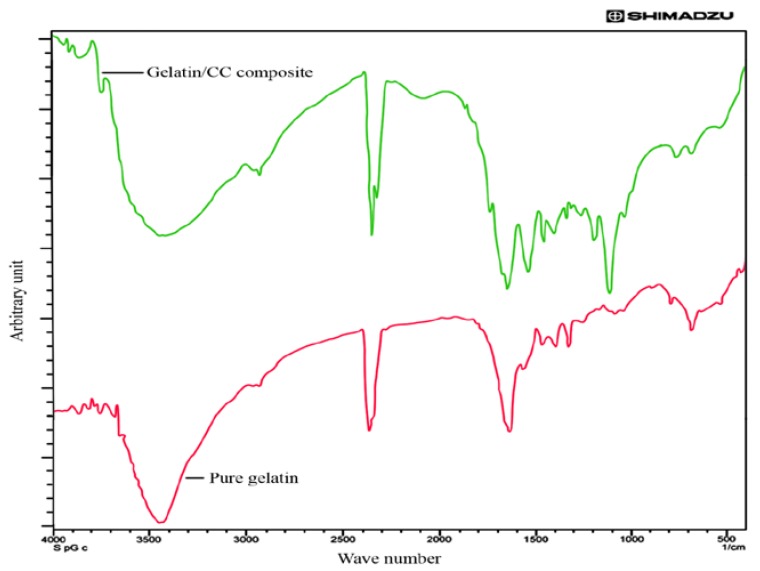
FTIR spectra of pure gelatin and gelatin/CCs biocomposite film.

FTIR is of importance in the study of molecular structure. Environmental changes and the conformation of macromolecules lead to changes in the intensity and the width of the spectrum bands, as well as the arrangement of the peaks. Figure 2 represents the FTIR spectrum of gelatin/CCs biocomposite compared with the spectrum of pure gelatin. FTIR spectrum of the pure gelatin showed that the peaks at 3450 and 3423 cm^−1^ were due to N–H stretching of secondary amide, C=O stretching at 1680 and 1640 cm^−1^, N–H bending between 1550 and 1500 cm^−1^, N–H out of plane wagging at 670 cm^−1^ and C–H stretching at 922 and 2850 cm^−1^. The characteristic peak of the collagen fold in 3360 cm^−1^ is absent in the spectrum, which indicates the denaturation of collagen to produce gelatin.

On the other hand, it was found that a characteristic peak that ranges from 1700 to 1800 cm^−1^ developed in the FTIR spectrum of Gelatin/CC blend film which indicated the presence of a hydroxyl group with polymeric association and a secondary amide.

It is estimated that the shortened bond length due to esterification leads to the shifting of the peak to a higher wavenumber. In the presented data the production of the esterified product was confirmed by the shifting of the peak at 1968.3 cm^−1^ of gelatin. Therefore, it can be concluded that the free carboxylic groups of gelatin have been esterified.

A broad peak was also found at 3200–3600 cm^−1^, which is the corresponding peak of hydrogen bonding between the NH of gelatin and OH groups of cellulose. Other interactions between gelatin and cellulose were also confirmed due to the formation of the peaks at the range of 1200–1600 cm^−1^.

### 3.3. Mechanical Properties Analysis

**Figure 3:**
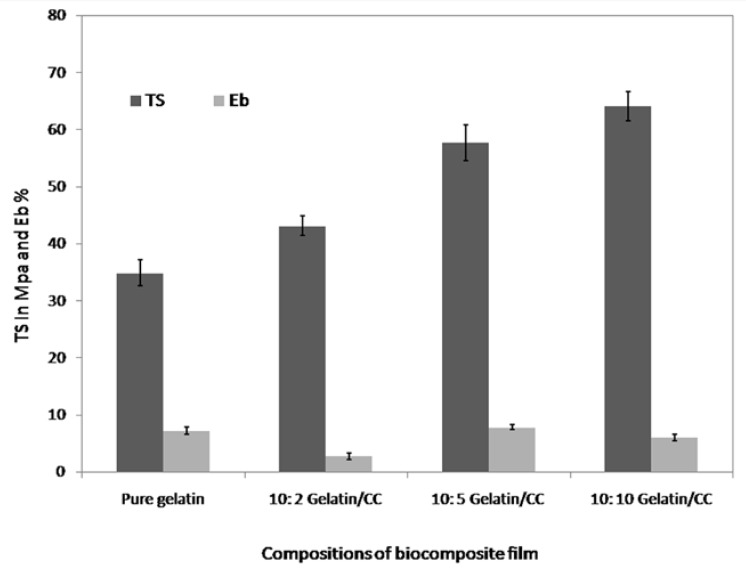
Comparison of the tensile strength and elongation at break of gelatin and gelatin/CCs biocomposite films.

Mechanical testing provides an indication of the strength and elasticity of the membrane, which can be reflected by tensile strength, elongation at break, and elongation modulus. The biocomposites were prepared using 2 mL, 5 mL, and 10 mL of CCs stock solution (as prepared) in 10 g of gelatin. Tensile strength (TS) and elongation at break (Eb%) of pure gelatin and gelatin/CCs biocomposite are graphically demonstrated in Figure 3. The average TS of the pure gelatin films were found to be 34.92 MPa. The average TS of the gelatin/CCs blend biocomposites at ratios 10:2, 10:5, and 10:10 were found to be 43.16, 57.74, and 64.16 MPa, respectively, while the TS for biocomposite of gelatin with other additives were about 20 MPa [39,40]; this indicates that our prepared biocomposite (gelatin/CCs) has higher resistance capability against breaking under tension than previously invented biocomposites. Moreover, it was observed from figure that gelatin/CCs blend biocomposite film at a 10:10 ratio showed 83.73% TS higher value than pure gelatin film. The ultimate tensile strength of the blends showed an increasing tendency with increase in percentage of CCs in gelatin within the range of the concentration studied.

On the other hand, the Eb did not show any significant increasing or decreasing trend throughout the gelatin CCs composition.

**Figure 4:**
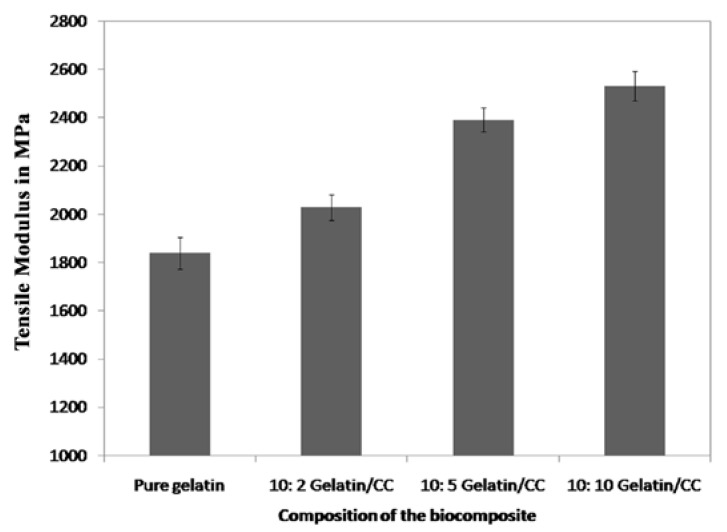
Comparison of the tensile modulus of gelatin/CC biocomposite films.

In the study TM (tensile modulus) values of gelatin-based films was improved significantly with the incorporation of CCs and the effect of CCs on the TM values of gelatin-based films is represented in Figure 4. For 10:2, 10:5, and 10:10 gelatin/CCs composite, the average TM values were observed to be 2.17, 2.42, and 2.64 GPa, respectively.

The amino group of the gelatin polypeptide chains acts as an electron donor and the hydrogen of cellulose as an electron acceptor [33], and the hydrogen of CCs have been suggested to form a hydrogen bond with the amino group, which induces dipole-dipole traction between two different phases, which is supposed to enhance molecular interaction and also for increasing affinity of the mechanical properties of CNCs-gelatin. The 10:10 gelatin/CCs film was considered as the optimum because the films have good strength (64.16 MPa), modulus (2.6 GPa), and suitable Eb% (6.05%). Moreover, the appearance of the films was quite transparent.

### 3.4. Fluid Drainage Properties

#### 3.4.1. Buffer Uptake

**Figure 5:**
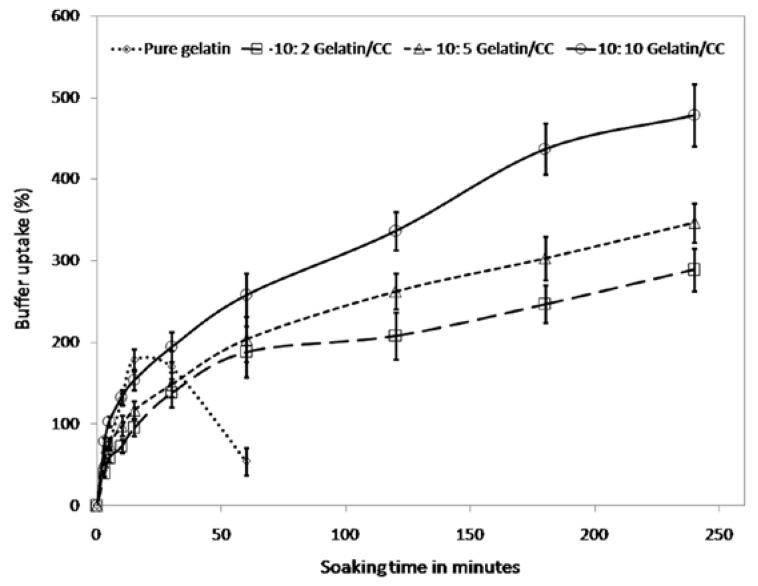
Comparison of buffer uptake of gelatin/CCs biocomposite films.

The fluid-absorbing capacity of a wound dressing material is a significant factor for maintaining a moist environment over the wound bed [36]. The phosphate buffer uptake of biocomposite films were studied for 5 h and plotted against time in Figure 5. The buffer uptake increased significantly with incorporation of CCs. The pure gelatin film could not sustain more than one hour in buffer, whereas the CCs-incorporated films showed increasing trend up to 5 h, although the slope of the curves decreased after 4 h of soaking.

This increased buffer uptake supports the findings of FTIR study. It can be assumed that there are strong hydrogen bonds between the C=O, –NH_2_ groups of gelatin, and –OH groups of CCs. There is another possibility of forming C–N bonds between the NH_2_ groups of gelatin and –OH–C groups of CCs leaving one molecule of water. In fact, these chemical and physical bondings/crosslinking are the reasons behind the increased hydrostability and gradual buffer uptake of the developed samples [41]. Thus, these results revealed that the developed gelatin/CCs biocomposite will be very suitable as advanced wound dressing material because they comprises appropriate properties to absorb wound exudates and, thus, will prevent wound from accumulating fluid. For assessing the mechanical and fluid drainage properties we used 10:10 gelatin/CCs film for further experiments.

#### 3.4.2. In Vitro Biocompatibility Analysis

**Figure 6:**
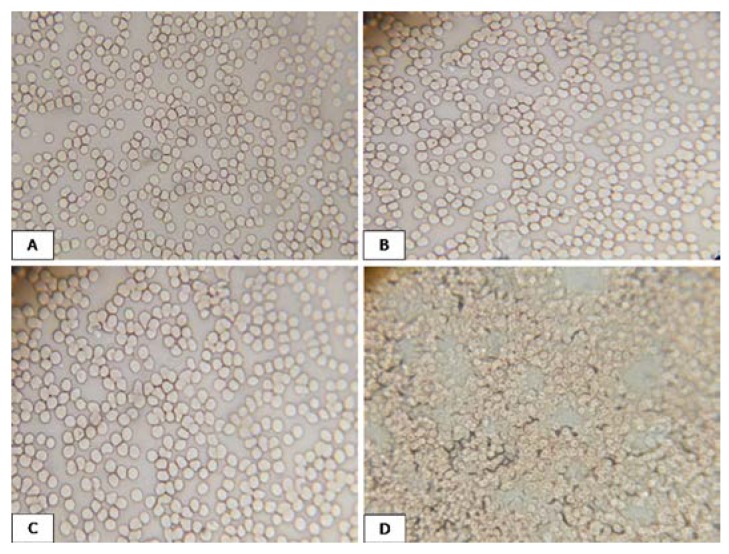
Microscopic view of (**A**) pure blood; (**B**) blood incubated in 2 (blood):1 ratio (biocomposite 10:10); (**C**) blood incubated in a saline solution in the same ratio; and (**D**) blood incubated in distilled water in the same ratio.

Light microscopic analysis was carried out to observe the red blood cell morphology by magnifying the designed slides forty items. RBC undergoes lysis or coagulation in contact with a nonbiocompatible agent. Our studies showed less than 5% hemolysis and the red blood cells remained intact when incubated with CCs biocomposite: blood in a 1:2 ratio indicated biocompatibility of the composite film (Figure 6). Similar results were found in the case of saline water incubation. On the other hand, cell damage occurred by distilled water incubation with the same ratio, which is due to osmotic shock and gave us an idea of the morphology of RBC in contact with a nonbiocompatible agent.

#### 3.4.3. Microbial Sensitivity Analysis

**Figure 7:**
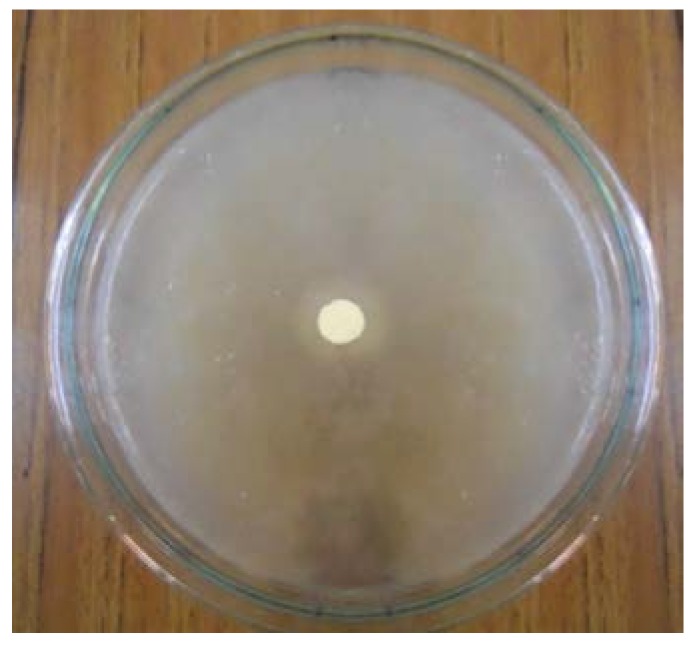
Bacterial growth in agar medium.

The experimental results suggested that the prepared biocomposite film (gelatin:CCs = 1:1) had no bactericidal effect on the chosen bacterial strain. Figure 7 shows no clear zone of inhibition on the agar plate. This finding predicted that the wound dressing with the biocomposites could not achieve a direct antimicrobial effect against *Pseudomonas* sp. which is the main cause of infection in the wounded area but it could prevent infection by inhibiting bacterial migration and penetration.

#### 3.4.4. In Vitro Cytotoxicity Test

The brine shrimp lethality bioassay method was used for understanding cytotoxic effect of the gelatin/CCs biocomposite film. The biocomposite film was dissolved in artificial sea water in which nauplii (larvae of brine shrimp) were inoculated. The results suggested that the biocomposite film induces death of the nauplii to some extent at the high dose (Table 1). This may occur due to three reasons: cytotoxic effect of the biocomposite, decrease of dissolved oxygen concentration of the saline water, and formation of the viscous layer on the gills of nauplii. In the present study, the possibility of death of nauplii owing to toxicity is very low as the number of deaths was nil for lower concentrations, suggesting no cytotoxic effect. Moreover, cellulose and gelatin were used as parent materials of the composite which were both biocompatible. Hence, the most possible reason for the death of nauplii is (i) the formation of the viscous layer on the gills of the nauplii as the highly-concentrated solution of the gelatin-based composite film led to high viscosity; and (ii) the formation of a gel-like structure on the gills which eventually inhibits oxygen permeability.

#### 3.4.5. In Vivo Wound Healing

**Figure 8:**
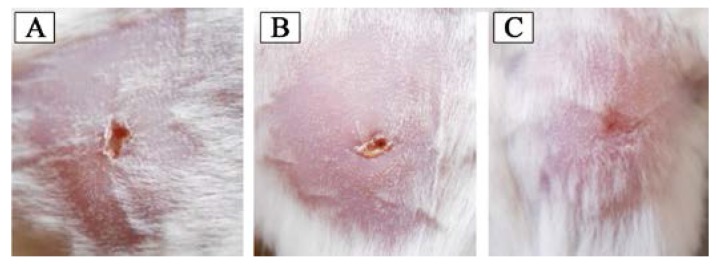
Wound healing of the experimental mice after 10 days of surgery; (**A**) dressed by conventional dressing; (**B**) dressed by pure gelatin film; and (**C**) dressed by the developed biocomposite film.

The performances of the developed biocomposite films as wound-healing materials were evaluated in an experimental mice model (Figure 8). No significant weight loss or fever was found during the total healing process. In the tenth day post-surgery, the biocomposite dressing was removed from the wound surface without having any further trauma and the wound surface was found almost totally healed and characterized with wound site contraction and re-epithelialization. The mice models dressed with the conventional dressing suffered from bleeding during removal of bandage (Figure 8). As a result, a delayed healing was observed. The pure gelatin film also showed better wound healing compared to the conventional dressing but the healing rate was lower than that of the biocomposite dressing. The edges of the wounds were found pulling inwards to reduce the overall wound area after 10 days of surgery.

## 4. Conclusions

Gelatin-CNCs composite was prepared by a solution casting method. CNCs were used as cross-linkers in order to stabilize gelatin by establishing cross-links between the protein chains. The results indicate the mechanical properties such as TS, TM, and Eb of the pure gelatin film are poor, while the enhancement of TS, TM, and Eb of the gelatin-CNCs films is sufficient. Moreover, water and buffer uptake properties of gelatin are efficiently improved due to the addition of CNCs. Furthermore, the composites are bio-compatible and non-toxic. Additionally, the gelatin-CNCs biocomposite for use as wound dressings is very effective since they can absorb wound exudates and provide a perfect moist environment for a healing wound. Prior researchers also reported that a moist wound environment is ideal for healing chronic wounds [42,43]. These findings will broaden the biomedical applications of the gelatin-CNCs biocomposites in wound dressings, tissue engineering, and sustained-release applications.

## Figures and Tables

**Table 1:** Amount and percentage of mortality of nauplii after the cytotoxicity test.

Sample No.	Sample Name	Dose (mg/mL)	No. of Nauplii Present after Incubation	Mortality (%)
1.	Positive control (vincristine sulfate)	0.5	0	100
2.	Negative control (artificial sea water)	-	10	0
3.	Biocomposite film	0.125	10	0
4.	Biocomposite film	0.25	10	0
5.	Biocomposite film	0.5	9	10
6.	Biocomposite film	0.75	8	20
7.	Biocomposite film	1.00	8	20
